# Aqueous Dilution of Noble NPs Bulk Dispersions: Modeling Instability due to Dissolution by AF4 and Stablishing Considerations for Plasmonic Assays

**DOI:** 10.3390/nano10091802

**Published:** 2020-09-10

**Authors:** Lorenzo Sanjuan-Navarro, Aaron Boughbina-Portolés, Yolanda Moliner-Martínez, Pilar Campíns-Falcó

**Affiliations:** MINTOTA Research Group, Departament de Química Analítica, Facultat de Química, Universitat de Valencia, 46100 Burjassot, Spain; lorenzo.sanjuan@uv.es (L.S.-N.); abough@alumni.uv.es (A.B.-P.); yolanda.moliner@uv.es (Y.M.-M.)

**Keywords:** AuNPs, AgNPs, asymmetrical flow field flow fractionation, stability, dispersion, dissolution, plasmonic assays

## Abstract

Among different nanomaterials, gold and silver nanoparticles (AuNPs and AgNPs) have become useful tools for a wide variety of applications in general, and particularly for plasmonic assays. Particle size and stability analysis are key elements for their practical applications since the NPs properties depend on these parameters. Hence, in the present work, asymmetrical flow field flow fractionation (AF4) coupled to UV-Vis and dynamic light scattering (DLS) detectors in series, has been evaluated for stability studies of citrate-capped AuNPs and AgNPs aqueous dispersions. First, experimental parameters, such as mobile phase or cross-flow rate were optimized. Sodium azide to pH 7 for AuNPs and pH 9.2 for AgNPs were selected as the optimum mobile phase. The analytical response of bulk dispersions of AuNPs (20, 40, 60 and 80 nm) and AgNPs (20, 40 and 60 nm) and their dilutions have been studied. Fractograms showed a decrease on the absorbance signal in diluted dispersions as a function of time and particle size for the diluted dispersions that can be explained by dissolution in diluted dispersion since hydrodynamic diameter was constant. The results indicated that the dependence of the signal with time was more intense for AgNPs than for AuNPs, which can be correlated with its lower stability. These findings should be considered when plasmonic assays are realized. Here, assays involving non-oxidant acidic acids as use cases, were tested for several batches of NPs and considerations about their stability and operability stablished.

## 1. Introduction

Nanoparticles and nanotechnology have a large socio-economic impact in research and in several industrial activity areas. Specifically, gold and silver nanoparticles (AuNPs and AgNPs) are now used in many of the fields of science, analytical applications [1], medical applications [2], bioimaging [3], construction industry and sensor technologies [4]. Therefore, their study and characterization are necessary to fully understand and monitor their properties [5]. Different techniques have been used to characterize and quantify NPs in order to stablish their physical and chemical properties. Counting techniques, optical techniques and separation techniques have been proposed to supply the chemical and physical information required in different fields [6,7,8,9,10].

**Figure 1:**
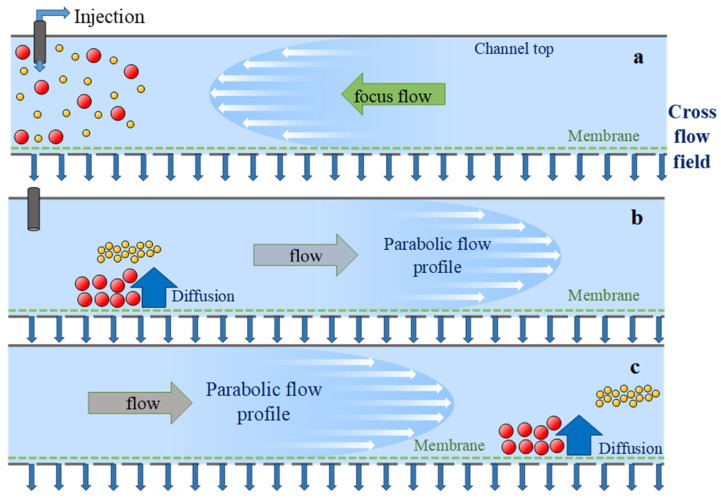
Schematic on the working principle of AF4: (**a**) Injection and sample focusing, (**b**,**c**) sample fractionation.

Asymmetrical flow field flow fractionation (AF4) is a continuous separation technique for particles and macromolecules that is based on hydrodynamic principles without using a stationary phase [11]. In this technique, a liquid cross-flow perpendicular to the channel drags the solute particles toward the accumulation wall and results in a size gradient. Diffusion back to the region of lower size acts as a counteracting force. These two counteracting forces balance each other and consequently a steady state distribution of analyte is established with the highest size at the accumulation wall that decreases toward the center of the channel [12]. Figure 1 shows the main steps involved in AF4: sample injection in the separation channel and sample focusing by a reverse flow (Figure 1a), and after fractionation (Figure 1b,c).

In particular, the metallic NPs transformations that can take place in dispersions must be considered in order to ensure the proper performance of these nanoparticles [13]. Aggregation, agglomeration, dissolution, reaction, or replacement of surface capping are potential transformations that can alter the performance and stability of metallic NPs [14,15].

Hence, in the present work, the dilution-induced variation of AuNPs and AgNPs were evaluated as a function of the dilution ratio, time, particle size, and batch. For this aim, AF4 on-line coupled with UV-Vis and DLS detector was used. Citrate-capped AuNPs and citrate-capped AgNPs with different particle size have been studied as a function of time. The main objective was to stablish the stability profiles, transformation mechanisms and therefore, the real performance of these nanomaterials for potential practical application in general and for plasmonic assays particularly. Guidelines for realizing plasmonic assays properly have been proposed. Here, plasmonic assays for hydrochloric and acetic acids are shown.

## 2. Materials and Methods

### 2.1. Reagents

AuNPs in citrate buffer solutions (average core diameter: 20 (18–22), 40 (37–43), 60 (57–63) and 80 (77–83) nm), AgNPs in citrate buffer solutions (average core diameter: 20 (16–24), 40 (36–44) and 60 (52–68) nm and 20 (18–22) nm) AuNPs in 0.1 mM phosphate buffered saline (PBS) (Sigma-Aldrich, Saint Louis, MO, USA) were used, all of them 0.02 mg/mL. Hydrochloric acid (37% *w*/*w*) and concentrated acetic acid were supplied by Sigma-Aldrich and VWR Chemicals (Radnor, PA, USA), respectively.

The liquid carrier for AF4 was prepared with sodium azide 0.02% (NaN3, Panreac, Castellar del Vallés, Barcelona, Spain) and pH was adjusted using 0.1 M sodium hydroxide (NaOH, Panreac). Methanol (VWR) was used for cleaning AF4 system.

3,3′,5,5′-Tetramethylbenzidine (TMB) (Sigma-Aldrich) and sodium acetate anhydrous (Panreac) were also used. Moreover, it was used silver nitrate (VWR) and gold (III) chloride trihydrate (Sigma-Aldrich). 

The water for all the experiments was purified through a Barnstead Nanopure II system.

### 2.2. Instrumentation

An AF2000 MT model was used to perform the AF4 measurements supplied by Postnova Analytics Inc. (Landsberg am Lech, Germany). The channel was 29 cm long with a 10 kDa regenerated cellulose membrane and 350 µm channel spacer. The flows were provided by two separate pumps and the cross-flow was got by a separate piston pump, which is constantly adjustable. For all AF4 analysis, the liquid carrier was high purity Mili-Q water containing 0.02% sodium azide, for AuNPs to pH 7 and for AgNPs top H 9.2. Samples were injected using an autosampler (Postnova, Germany) and the injection volume was 20 µl. Optimal separation was achieved using the conditions explained in S.1 of the Supporting Information (SI) for each NPs and the flow was kept at 0.5 mL/min. 

The AF4 system was coupled online with a UV-Vis detector (SPD-20AV, Postnova, Germany) and a DLS detector with temperature control (Nano-ZS, Malvern, UK). The UV-Vis detector was operated at the wavelength of 530 nm to AuNPs and in the interval 395–410 nm to detect AgNPs. For DLS detection, AF4 system was directly interfaced to a Zetasizer without channel split and the flow was set to 0.5 mL/min for all fractions.

Transmission Electron Microscope (TEM) samples were prepared by delivering 10 µL of the NP solution onto carbon-coated copper grid (300 mesh) and was dried overnight at room temperature. These samples were analyzed by a JEM1010 from Jeol Ltd (Akishima, Tokyo, Japan). operated at 100 kV. An Agilent (Santa Clara, CA, USA) Cary 60 UV-Vis spectrophotometer was also employed for spectroscopy studies.

### 2.3. TMB Assay

A colorimetric assay, previously reported by our group [16], was carried out to evaluate the presence of Au or Ag ions in the dispersion. 1.2 mL of NPs dilution dispersion was mixed with 100 µL of TMB 10 mM in EtOH and 200 µL of NaAc/HAc buffer 1 M, pH = 4. After 15 min, the mixture changed a blue color complex that indicated the presence of these ions [16].

### 2.4. Plasmonic Assays

Several batches of AgNPs were diluted with ultrapure water in 1:4 proportion containing concentrations of acetic acid between 1.5 and 15 mM (pH ≈ 3–4) or hydrochloric acid between 0.1 and 15 mM (pH ≈ 2–4). The several dispersions were measured by UV-Vis spectroscopy and AF4.

## 3. Results and Discussion

### 3.1. Characterization of Citrate-Capped—AuNPs and AgNPs Dispersions

A study of the mass response of the AF4 was carried out for both types of NPs, the same peak areas were obtained by processing 5, 10 and 20 µL of diluted dispersions 1:2, 1:4 and 1:8 of ultrapure water, respectively; the area values were 2.5 ± 0.3 (*n* = 10) and 8.0 ± 0.2 (*n* = 8) vs. for AuNPs and AgNPs, respectively. The injection volume selected for processing samples in AF4 was 20 µL.

Aqueous dilution of commercial citrate-capped AuNPs and AgNPs of several sizes (20, 40, 60 and 80 nm for AuNPs and 20, 40 and 60 nm for AgNPs) and at different dilution ratios were studied.

**Figure 2:**
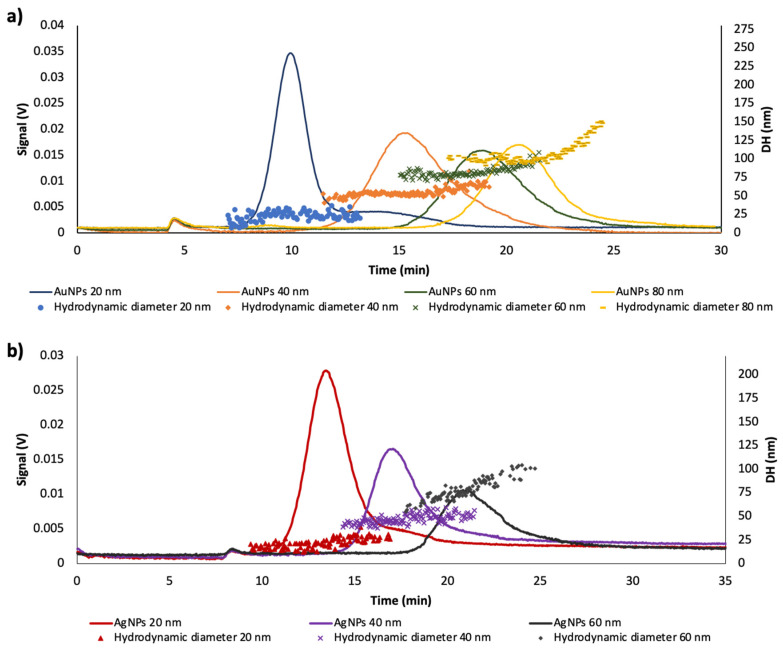
Fractograms for: (**a**) 20, 40, 60 and 80 nm citrate-capped-AuNPs aqueous dispersions (dilution: 1/4) and (**b**) 20, 40 and 60 citrate-capped-AgNPs aqueous dispersions (dilution: 1/4).

Figure 2a shows the fractograms obtained for AuNPs with different sizes at the same dilution ratio (1/4), injected just after its preparation, under the experimental conditions described in Section 2. It should be noted that the retention time is directly related with the nanoparticle size, larger nanoparticles experimented a greater retention into the channel as result of their minor diffusion coefficient [17]. The peak that appears at ≈5 min corresponded to the void peak.

The correlation between the peak area and dilution ratio (between 1/2 to 1/10) for each size were studied. Table 1 depicts the relationship between the response obtained with the UV-Vis detector and the dilution ratio. As it was expected, a lineal correlation was observed for all AuNPs sizes with satisfactory regression coefficients (R^2^ = 0.999–0.991). Moreover, the slope, and thereof the sensitivity was higher for the smaller AuNPs at the working wavelength (520 nm).

Precision studies were also performed, for this aim relative standard deviation (%RSD) was calculated in order to determine the stability of bulk dispersion for each NPs size. In this assay, four different AuNPs dispersions were prepared following the same procedure and immediately analyzed in the AF4 system. The results showed that RSD values were lower than 6.5%, and therefore, intraday precision was suitable in aqueous diluted dispersions.

The same study was carried out for AgNPs (20, 40, 60 nm). Figure 2b shows the fractograms of these nanoparticles. The peak areas as a function of the particle size and RSD values for citrate-capped AgNPs were also calculated (Table 1). Precision was lower than that achieved by AuNPs since the RSD values were higher, near 10%, although suitable for general applications, which can show the lower AgNPs’ stability.

AuNPs’ and AgNPs’ recoveries from the AF4 channel were also calculated. Values between 10.3 and 29.4% for AuNPs, and between 31.6 and 65.8% for AgNPs were found. These results are in agreement with previous reports, which demonstrated that the interactions between the membrane and NPs have a high probability of undergoing irreversible interactions [18,19].

### 3.2. Effect of Time in the Analytical Responses of Diluted Dispersions of NPs

**Figure 3:**
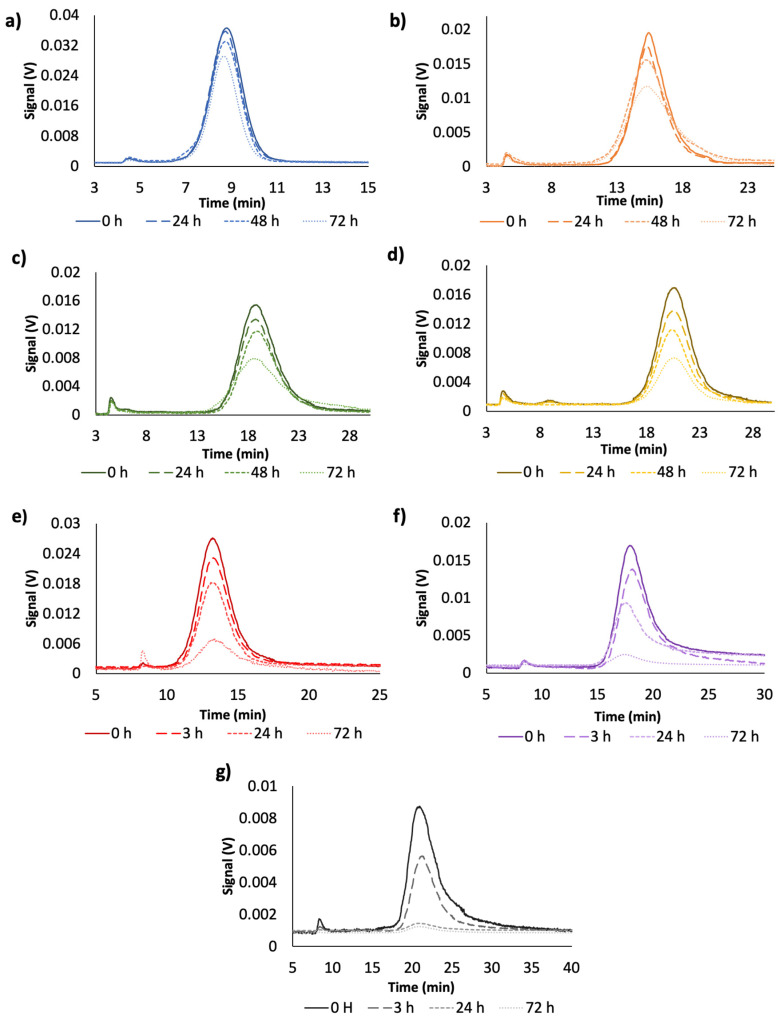
Fractograms of the evolution of nanoparticles dispersions as function of dilution preparation time for different sizes (**a**) AuNPs 20 nm, (**b**) AuNPs 40 nm, (**c**) AuNPs 60 nm, (**d**) AuNPs 80 nm, (**e**) AgNPs 20 nm, (**f**) AgNPs 40 nm and (**g**) AgNPs 60 nm.

**Figure 4:**
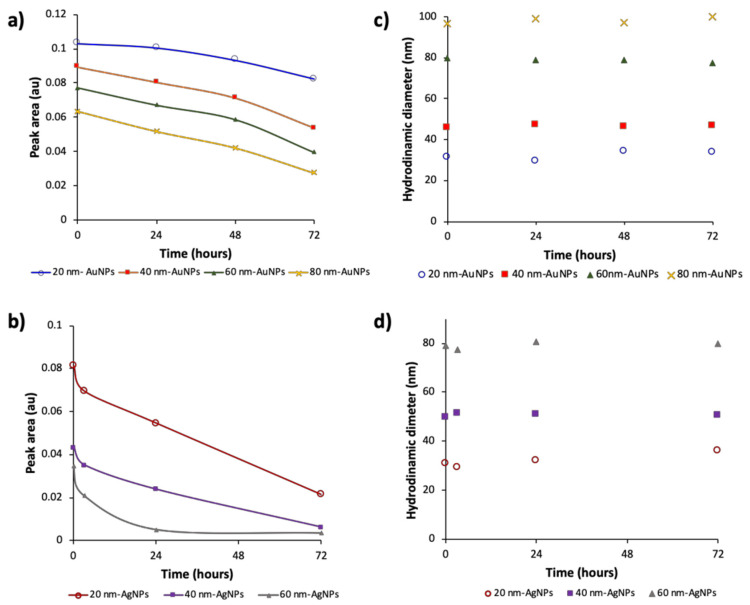
(**a**) Variation of the peak area as a function of time for citrate-capped-AuNPs. (**b**) Variation of the hydrodynamic diameter as a function of time for citrate-capped-AuNPs. (**c**) Variation of the peak area as a function of time for citrate-capped-AgNPs and, (**d**) Variation of the hydrodynamic diameter as a function of time for citrate-capped AgNPs.

The environment of metallic NPs is a key parameter for their practical application, since their performance will depend on their stability. In several application, dilution of these NPs are needed; however, dilution-induced changes, mainly on the surface interactions that may affect the performance of this NPs. These changes depend on the kinetic of individual NPs subject to local variations and thereof, the time is an important parameter. To prove this, citrate-capped AuNPs diluted dispersions (1/4) of different sizes were measured at different times after their dilution. Figure 3 shows the fractograms obtained at different times after dispersion preparations (t = 0, 24, 48 and 72 h). As can be seen in Figure 3a–d and Figure 4a, there was a decrease in the absorbance signal with time, and that decrease, depended on the particle size.

The hydrodynamic diameter of the different AuNPs was measured as a function of time with the DLS detector coupled on-line with the UV-Vis detector. Figure 4b shows the results obtained for the different sized NPs. As can be seen, the hydrodynamic diameter was constant over the dilution, in addition it was constant with the time and this effect was observed for all NPs sizes (DLS graphics can be seen in S.2 of the SI).

The same study was carried out for citrate-capped AgNPs in order to check whether the nature of NPs had some influence on the previous results. Figure 3e–g represents the signal variation for dispersions with different sizes (20, 40 and 60 nm). As in the case of citrate-capped AuNPs, there was a decrease on the signal with time, and that decrease was a function of the particle size. Indeed, the analytical response for 60 nm AgNPs at 24 h was negligible.

However, compared with AuNPs, the increment of that decrease was higher for AgNPs (see Figure 4b), which is consistent with the higher stability of AuNPs than that given by AgNPs [20,21]. Figure 4c,d shows the variation of the hydrodynamic diameter for both NPs being constant with time for both of them. A diluted aqueous dispersion of AuNPs in PBS (dilution 1/8) was measured as a function of time. As can be noted in the Supporting Information (S.3), it was observed the same effect that in previous assays for citrate-capped NPs.

**Figure 5:**
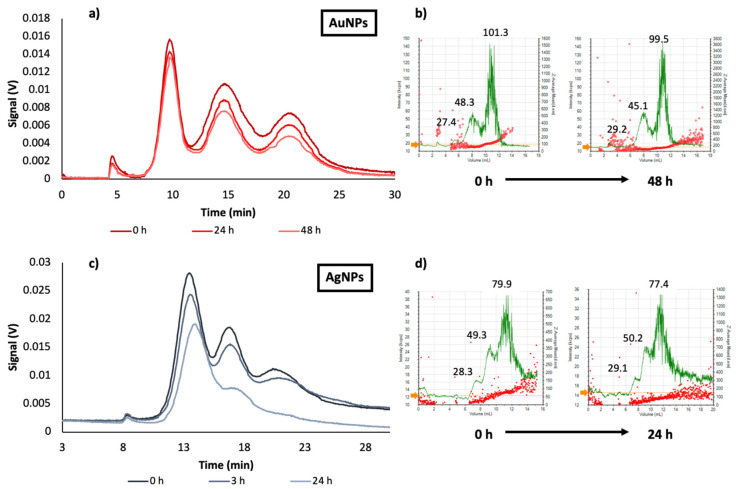
Mixtures of different sizes of AuNPs (dilution 1/8) and AgNPs (dilution 1/4). (**a**) Fractograms of the evolution of 20, 40 and 80 nm AuNPs mixture; (**b**) DLS values of AuNPs sizes, DLS fractograms in green and hydrodynamic diameters distribution in red; (**c**) fractograms of the evolution of 20, 40 and 60 nm AgNPs evolution mixture; (**d**) DLS values of AgNPs sizes, DLS fractograms in green and hydrodynamic diameters distribution in red.

Figure 5 shows the evolution of signal as function of dilution preparation time for mixtures of different sizes of AuNPs with dilution (1/8) and AgNPs with dilution (1/4). As it was expected, fractograms of citrate-capped AuNPs mixture (20, 40 and 80 nm) were resolved, and three peaks were obtained corresponding to 20, 40 and 80 nm AuNPs. As above mentioned, 24 h after the dilution preparation, the UV-Vis signal changed as a function of the particle size, in accordance with the results obtained for individual NPs; NPs with higher diameter experienced a decrease on the peak area higher than that provided by smaller NPs. This effect was more drastic after 48 h as it can be seen in Figure 5a.

In the case of the AgNPs mixture (20, 40, and 60 nm) the same evolution that that described for AuNPs was observed (Figure 5c). However, the lower stability of AgNPs due to interactions with medium, produced a more pronounced signal reduction, since dissolution in this case was more favored than for AuNPs. Indeed, the peak for 60 nm citrate-AgNPs was not detected after 24 h. The average hydrodynamic diameters of dispersed NPs were stable during the whole analysis (see Figure 5b,d), as previously stated for individual particles. These results could support the hypothesis that dissolution is the mechanism by which the stability of dispersed NPs in diluted dispersions varied as a function of time.

**Figure 6:**
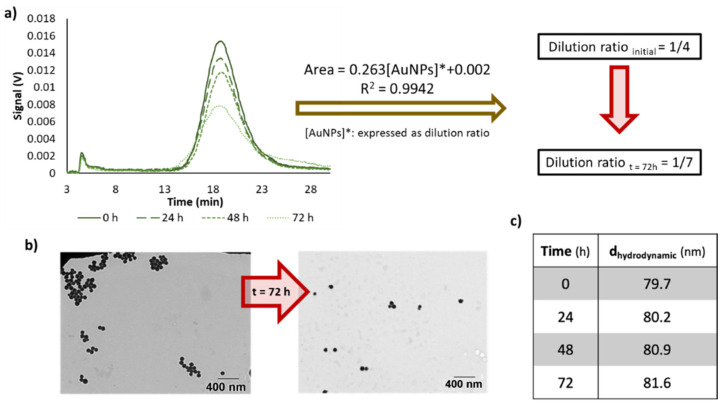
(**a**) Effect in the dilution ratio for AuNPs 60 nm with time. (**b**) TEM micrographs for diluted dispersions of citrate-capped-AuNPs (60 nm) just after their preparation and after 72 h. (**c**) Hydrodynamic diameter of AuNPs at different dilution times (0, 24, 48 and 72 h).

Figure 6 summarizes the changes for 60 nm AuNPs as an example. As can be seen, there is a decrease on the signal, and after 72 h, the dilution ratio varied from 1/4 to 1/7, in the case of this NPs (Figure 6a), but its size was constant (Figure 6c). TEM analysis showed that the NPs core size did not vary (Figure 6b). Similar considerations can be done for the other NPs analyzed.

**Figure 7:**
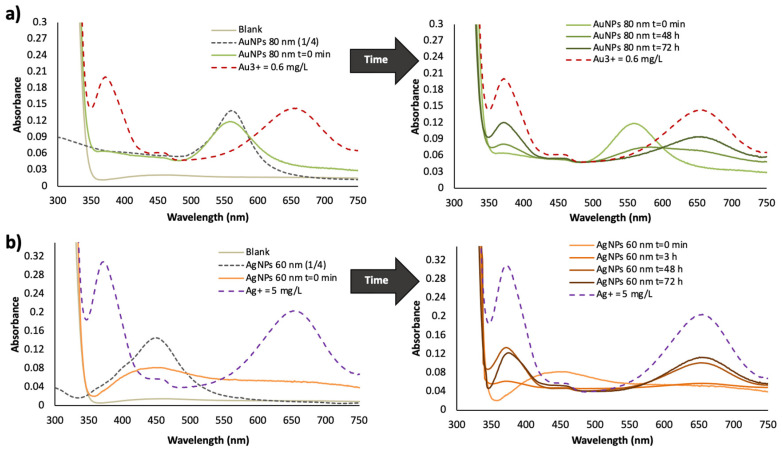
Gold and silver nanoparticles dissolution TMB assay for several times of preparation of diluted dispersions from batch NPs: (**a**) AuNPs for 0, 48 and 72 h and (**b**) 0, 3, 48 and 72 h. For more explanation see text.

A TMB assay [16] was applied in order to corroborate the instability by dissolution of the dispersions. In this assay, Au and Ag ions can be detected in NPs dispersions, since the cationic species reacts with TMB to form a blue color compound (λmax = 650 nm). The first spectra of Figure 7a,b show those obtained for just prepared diluted dispersions of AuNPs and AgNPs before and after the reaction with TMB, respectively. These spectra were compared with the spectra obtained for Au^3+^ and Ag^+^ standards after TMB derivatization as control experiments and blank solution of the TMB assay. As can be seen, cationic species were not detected for recently diluted dispersions, since there was not band at 650 nm, and only their plasmon bands were observed. Several diluted dispersions of NPs were prepared from the batch NPs, and processed by the TMB assay after different preparation times (see Figure 7a,b). The absorbance at 650 nm increased with the dispersion dilution time, which is correlated with the presence of Au^3+^ and Ag^+^, indicative of dissolution of NPs.

Figure 7b shows the spectra for AgNPs, which indicates that the dissolution of this type of NPs is bigger than that achieved by AuNPs (see Figure 7a). This assay supported also dissolution as the reason of the loss of NPs with time for diluted dispersions.

From these results, changes of AuNPs and AgNPs with time must be taken into account since the dispersed fraction of NPs available in diluted dispersions varies with time, and therefore practical application of NPs in general must consider this effect in order to obtain reliable results. Besides, the main analytical parameters in particular, can be wrong if diluted dispersion are not deeply understood. If the fraction of NPs in diluted dispersions changes, for example limit of detection (LOD) will vary as a function of time, and this must be taken into account in order to characterize an analytical method. To demonstrate this, Table 2 shows the variation in the detection limits calculated for the diluted dispersions used after 72 h of its preparation, and these values are compared with the LOD calculated for diluted dispersions just prepared from the batch material. LOD was calculated by establishing the dilution ratio of a dispersion providing a signal corresponding to three times the instrumental noise from the equation signal vs. dilution ratio for each size, and taking into account the initial concentration.

### 3.3. Plasmonic Assays

**Figure 8:**
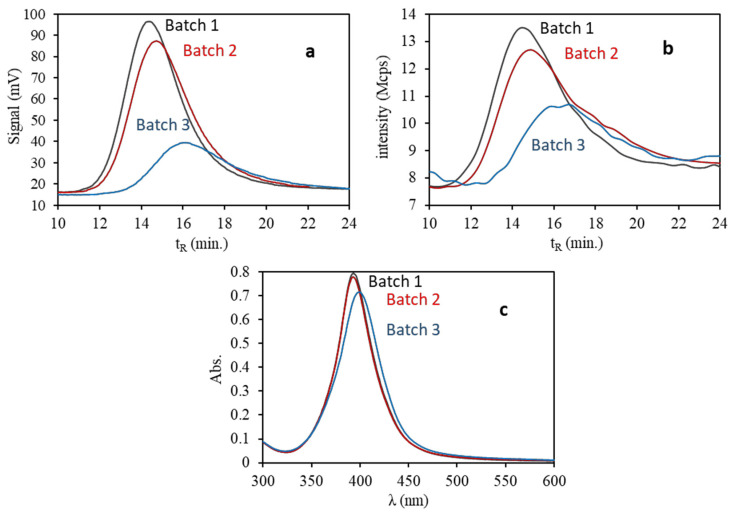
Fractograms of three different batches of AgNPs 20 nm diluted 1:4 with ultrapure water (5 µg/mL) with UV-vis detection (**a**) and DLS detection (**b**) and their spectra (**c**).

From the previous results concerning stability of the citrate—capped gold and silver NPs, we used freshly diluted dispersions for plasmonic assays. Three different batches of AgNPs 20 nm selected as a use case were assayed. Figure 8 shows the fractograms and their spectra, as it can be seen two of them (batches 1, 2) were similar, but the responses of the other (batch 3) were so different by AF4 with both UV-Vis and DLS detectors. A displacement of the retention time of the maximum of the peak is seen up to 16 min for batch 3, compatible with the presence of larger NPs, as well as a considerable decrease in their signal at λ = 395 nm, which can respond to a lower mass of AgNPs. In addition, the DLS data determine a mean hydrodynamic diameter around 2 nm greater than those of the rest of the batches, as shown in Table 3. By UV-Vis spectroscopy, all batches present a similar surface plasmon band (SPB), although batch 3 shows certain differences, since it provides somewhat lower absorption and a slight bathochromic shift, which could be consistent with slightly larger AgNPs.

The fractogram recorded using the DLS detector signal indicates a decrease in the intensity of the scattered light for batch 3 with respect to the rest (see Figure 8b), which signifies the presence of a smaller quantity of AgNPs. Phenomena such as dissolution and/or passivation could be considered to be a consequence of prolonged exposure to atmospheric conditions of batch 3 [22,23,24,25]. A part of the AgNPs could be dissolved and released in the form of Ag^+^, or it could be oxidized, generating a surface film of Ag2O, whose formation can be compatible by a bathochromic shift, as well as a lower height and broadening of the plasmon by UV-Vis spectroscopy (see Table 3 and Figure 8c) [22,24,25]. In this case, the main differences between batches could be related with the time that each of them has been exposed to the atmosphere, and consequently, with the renewal of dissolved oxygen in the suspension. If the AF4 records are compared with those obtained by UV-Vis spectroscopy, it should be noted that the AF4 technique shows a greater discrimination capacity in the characterization of aqueous dispersions of AgNPs, providing besides more information about mass and size.

**Figure 9:**
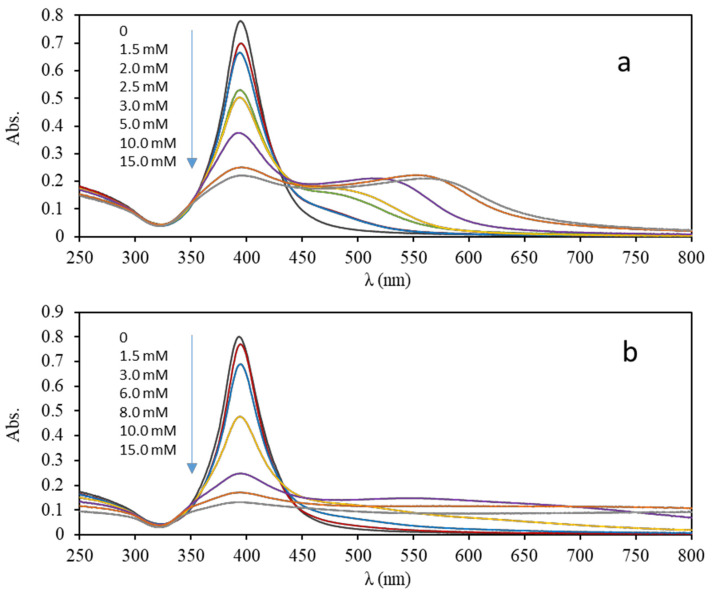
UV-Vis spectra recorded after 10 min. corresponding to the suspension of AgNPs in a 1: 4 dilution (5 μg/mL) in the presence of different concentrations of acetic acid (**a**) and hydrochloric acid (**b**).

We selected batch 1 for plasmonic assays involving hydrochloric and acetic acids. Figure 9 shows the spectra obtained by UV-Vis spectrometry corresponding to these assays at 10 min, which are compatible with aggregation processes. By decreasing the pH of the medium, a reduction in the height of the plasmon can be observed accompanied by an increase in the absorption at higher wavelengths until the formation of a second peak whose wavelength undergoes a bathochromic shift with time and with the concentration of acid used. For hydrochloric assay, the disappearance of this second peak is observed to give rise to a continuous band of absorption related with the formation of large-sized NPs that can be part of a polydisperse suspension of AgNPs of a wide range of sizes capable of scattering light from the visible spectrum and near-infrared range (NIR). The footprints of two acids were different, the changes for acetic acid are slower that those shown for hydrochloric acid by assaying the same concentrations (between 1.5 and 15 mM), which can be related by its acid-base strength.

**Figure 10:**
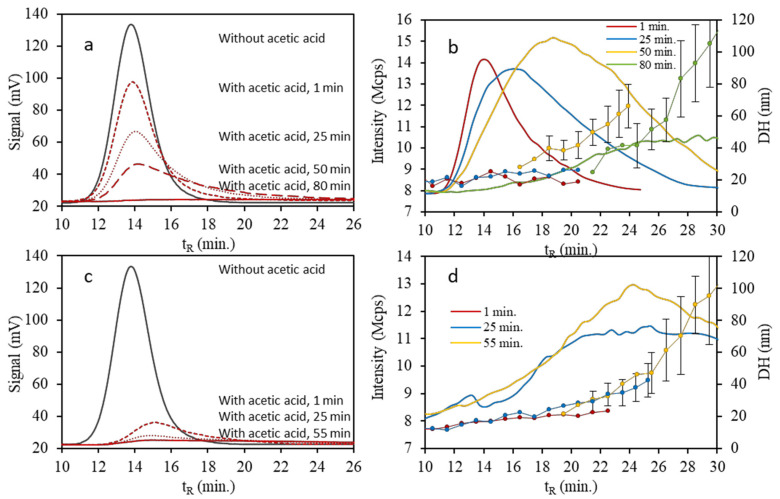
Fractograms wth UV-Vis detection corresponding to suspensions of AgNPs in a 1: 4 dilution (5 μg/mL) and 1.5 mM (**a**) or 3 mM (**c**) acetic acid at different times. Fractograms with DLS detection and hydrodynamic diameter (DH) (confidence interval α = 0.05) corresponding to the same suspensions at different times (**b**,**d**) respectively.

On the other hand, we studied the kinetics of the aggregation by AF4, Figure 10 shows the fractograms corresponding to the dispersion of AgNPs in the presence of 1.5 and 3.0 mM of acetic acid at several times since its preparation. The time evolution of the suspension containing 3.0 mM is more severe than that presented by the suspension containing 1.5 mM. By reducing the pH, a decrease in the height of the peak with time is observed in the fractograms recorded using the UV-Vis detector (Figure 10a,c), as well as a slight increase in retention times. The fractograms recorded using the DLS detector (Figure 10b,d) show a significant increase in retention time with time, as well as a slight increase in peak intensity, which indicate a shift in the size distribution of the AgNPs toward greater dimensions. These observations are compatible with aggregation of AgNPs, since there is a decrease in the intensity of the plasmon wavelength and a growth with time in the size of the NPs, as it can be seen in the hydrodynamic diameter data in Figure 10b,d. Likewise, a higher speed is observed, as well as a larger size of the aggregates when the pH decreases [26,27,28,29,30].

Under acidic pH conditions, the citrate capping, which provides stability to AgNPs, progressively loses its effectiveness by decreasing the surface negative charge density; the zeta potential in the interface that separates the Stern layer and the diffuse layer is less negative by reducing the pH of the medium [27,28]. NPs lose the repulsive forces that counteract the attractive van der Waals forces by removing the surface coating that protects them, thus facilitating their aggregation. It is observed with the exposure time that fractograms with a lower intensity of light scattering are recorded by the DLS detector as it can be seen in Figure 10b, which occurs more rapidly at lower pH (see Figure 10d). A greater tendency to oxidation of the NPs and release to the environment in the form of Ag^+^ as the pH decreases is also described [31]. However, it must also be taken into account that the aggregation process implies the formation of sets of NPs from others of a smaller size, which can lead to a significant reduction in their total number. Specifically, for the formation of 100 nm NPs dozens of 20 nm NPs may be necessary depending on the morphology of the final aggregates. This fact can also cause a decrease in the intensity of scattered light registered by the DLS detector when aggregates of size much larger than the initial NPs are formed. Thus, these aggregates can cause a greater scattering of light, but there are much fewer of them.

In this sense, the response of the AgNPs to the action of different acids, consists of the loss of protection against the aggregation process, which can be observed by the decrease and the bathochromic shift in the plasmon (SPB) using UV-Vis spectroscopy, related with the increase in retention times, loss of area and higher hydrodynamic diameter values showed by AF4-UV-Vis-DLS. Likewise, reducing the pH of the suspensions increased the aggregation speed, as well as it can facilitate the oxidation and /or dissolution of the AgNPs surface. These statements should be considered for developing plasmonic assays for quantifying analytes properly.

**Figure 11:**
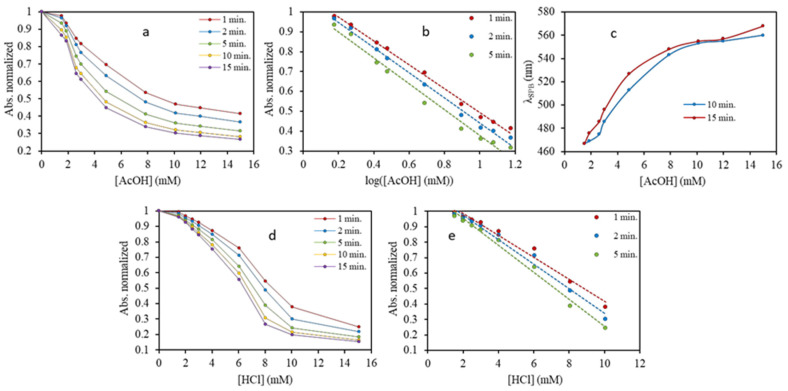
Normalized absorbance values at various times with the concentration of acetic acid (**a**), its log (**b**) of AgNPs diluted 1:4 (5 μg/mL) and evolution of the wavelength of the second plasmon maximum in the UV-Vis spectra at 10 and 15 min of its preparation (**c**). (**d**,**e**) correspond to the influence of hydrochloric acid.

Figure 11 shows the normalized absorbance values obtained by UV-Vis spectrometry at different times for each of assayed suspension against the concentration of acetic acid (11a) and hydrochloric acid (11d). A sinusoidal response is observed which for a certain range of concentrations, offers linearity in the change trend of absorbance with the acid concentration at a given time. This trend constitutes the basis on which plasmonic assays for the detection and / or quantification of substances are based [32,33]. Other possibilities are transforming the concentrations in their logs (see Figure 11b) or employing the maximum wavelength of the aggregated NPs for quantifying as it is done for acetic acid calibration (see Figure 11c). For all types of calibration graphs, the time influenced their equation and also the linear interval of concentrations (see Figure 11b,e), sensitivity and accuracy of the assay as can be reflected in Figure 11. Those aspects should be considered when the plasmonic assay is carried out.

Mainly, the application of AF4-UV-Vis-DLS provides new information about transformations that analytes induce in NPs and aid to fix the experimental variables, which must be controlled for obtaining reliable results. Besides, taking into account the great discrimination capacity of AF4 for explaining transformations generated in aqueous dispersions of NPs, their use could increase assay sensitivity if necessary. Please note that the dispersions assayed by AF4 corresponded to the lower concentrations tested by UV-Vis spectroscopy (see Figure 10 and Figure 11 for comparison). In this sense, stablishing the equation of the AF4 calibration graphs for plasmonic assays is outside the goal of this paper.

## 4. Conclusions

In this work, AF4 on-line coupled with UV-Vis and DLS detector was used to evaluate the impact of aqueous dilution on analytical responses of AuNPs and AgNPs dispersions. Different size dispersions were analyzed and the evolution of the fractograms profiles have been studied. The results demonstrated that for citrate-capped AuNPs and AgNPs, there was a decrease in the absorbance signal with time that depended on the particle size, this decrease was higher for bigger NPs. By another hand, hydrodynamic diameter was constant over dilution. AF4-UV-Vis-DLS showed that there is an effective variation in the number of NPs dispersed as a function of time, this variation was attributed to their dissolution in aqueous diluted dispersions. Comparing citrate-capped AgNPs and AuNPs, dissolution of the former NPs is more favored due to their lower stability, and the decrease on the absorbance signal as a function of time was more drastic. Hence, it can be concluded that AuNPs and AgNPs stability varied in diluted dispersions, and therefore their stability must be guarantee in order to obtain reliable results. In this context, AF4-UV-Vis-DLS demonstrated to be a potential tool to monitor variations in aqueous dispersions of metallic NPs, useful for understanding plasmonic assays and carrying out them properly. Other aspect to consider is the stability of the batches, it is demonstrated that the interaction of the batch with atmosphere modifies the number of NPs.

## Figures and Tables

**Table 1:** Values of RSD (%) and correlation between peak area and dilution ratio for AuNPs (20, 40, 60 and 80 nm) and AgNPs (20, 40 and 60 nm). A: ordinate and B: slope of the straight lines.

	Dilution Ratio	Concentration 10^2^ (mg/mL)	A ± SA	B ± SB (%^−1^)	R^2^	RSD (%)
AuNPs 20 nm	1/2 to 1/10	2.66 to 0.53	0.002 ± 0.004	0.409 ± 0.015	0.9961	2.2
AuNPs 40 nm	1/2 to 1/8	2.33 to 0.58	0.002 ± 0.003	0.338 ± 0.008	0.9988	5.3
AuNPs 60 nm	1/2 to 1/8	2.15 to 0.54	0.002 ± 0.005	0.262 ± 0.014	0.9942	6.3
AuNPs 80 nm	1/2 to 1/8	2.03 to 0.51	0.000 ± 0.005	0.233 ± 0.016	0.9911	6.4
AgNPs 20 nm	1/2 to 1/8	1.00 to 0.25	0.001 ± 0.005	0.339 ± 0.019	0.9904	3.1
AgNPs 40 nm	1/2 to 1/6	1.00 to 0.33	0.001 ± 0.004	0.171 ± 0.013	0.9883	9.9
AgNPs 60 nm	1/2 to 1/6	1.00 to 0.33	0.002 ± 0.003	0.119 ± 0.009	0.9890	10.1

**Table 2:** Detection limits calculated for each diluted dispersions of citrate-capped AuNPs and AgNPs.

		Co (mg/mL)	LOD (µg/mL) t = 0 min	LOD (µg/mL) t = 72
AuNPs	20 nm	0.053	0.22	0.26
	40 nm	0.047	0.12	0.17
	60 nm	0.043	0.14	0.21
	80 nm	0.041	0.06	0.09
AgNPs	20 nm	0.020	0.10	0.18
	40 nm	0.020	0.23	0.43
	60 nm	0.020	0.47	0.89

**Table 3:** Wavelength values (λ) of the plasmon maximum, width of the peak at half the maximum, extinction coefficient (εmax) obtained by UV-Vis spectroscopy and hydrodynamic diameter (DH) by DLS for three different commercial batches of AgNPs.

Batch	λ (nm)	Width_1/2_ (nm)	εmax (mM^−1^·cm^−1^)	DH ^a^ (nm)
3	397.6 ± 2.4	50.2 ± 0.1	15.43 ± 0.02	22.3 ± 2.4
2	393.4 ± 2.0	42.7 ± 0.2	16.83 ± 0.05	20.5 ± 3.1
1	393.1 ± 2.2	42.8 ± 0.2	17.05 ± 0.03	20.1 ± 3.0

^a^ Confidence interval α = 0.01.

## Supplementary Materials

The following are available online at https://www.mdpi.com/2079-4991/10/9/1802/s1, S.1.: Table S1. Method parameters for AuNPs. Table S2. AF4 method parameters for AgNPs. S.2.: Table S3. Hydrodynamic diameters for AuNPs dispersions as a function of dilution preparation time. Table S4. Hydrodynamic diameters for AgNPs dispersions as a function of dilution preparation. Figure S1. DLS spectra for AuNPs dispersions as a function of dilution preparation time. Figure S2. DLS spectra for AgNPs dispersions as a function of dilution preparation time. S.3.: Figure S3. Study of AuNPs-PBS dispersions as function of dilution preparation time.
