# Supplementary material for: Aqueous Dilution of Noble NPs Bulk Dispersions: Modeling Instability due to Dissolution by AF4 and Stablishing Considerations for Plasmonic Assays
Source: Nanomaterials (Basel). 2020 Sep 10;10(9):1802. doi: 10.3390/nano10091802 (PMC7560132; doi:10.3390/nano10091802)
Supplement: Supplementary file 1 [file nanomaterials-10-01802-s001.pdf]

nanomaterials

Supplementary Information

Aqueous Dilution of Noble NPs Bulk Dispersions: Modeling Instability due to Dissolution by AF4 and Stablishing Considerations for Plasmonic Assays

L. Sanjuan-Navarro, A. Boughbina-Portolés, Y. Moliner-Martínez and P. Campíns-Falcó *

MINTOTA Research Group, Departament de Química Analítica, Facultat de Química, Universitat de Valencia, 46100 Burjassot, Spain; lorenzo.sanjuan@uv.es (L.S.-N.); abough@alumni.uv.es (A.B-P.); yolanda.moliner@uv.es (Y.M.-M.)

* Correspondence: pilar.campins@uv.es; Tel.: +(34) 96 354-3002; Fax: +(34)96 354 3447

S.1. The AF4 system used was an AF2000 MT model purchased from Postnova Analytics Inc. (Germany). Optimal separation was achieved using the following conditions for each NPs.

Table S1. Method parameters for AuNPs.

<table>
<tr><td colspan="2">Eluent: Ultrapure water + 0.02% NaN_3; pH: 7</td></tr>
<tr><td colspan="2">Injection / Focusing
Detector flow rate (mL min^{-1}): 0.50
Injection flow rate (mL min^{-1}): 0.20
Focus flow rate (mL min^{-1}): 1.30
Cross flow rate (mL min^{-1}): 1.00
Injection time (min): 3.0
Transition time (min): 1.0</td></tr>
<tr><td>1st elution step
Elution time (min): 30.0
Elution type: linear
Exponent: 1
Initial cross flow (mL min^{-1}): 1.00</td><td>2nd elution step
Elution time (min): 10.0
Elution type: constant
Exponent: 0
Initial cross flow (mL min^{-1}): 0.00</td></tr>
</table>

Table S2. AF4 method parameters for AgNPs.

**Eluent:** Ultrapure water + 0.02% NaN_3; pH: 9.2	
**Injection / Focusing**<br>Detector flow rate (mL min^{-1}): 0.50<br>Injection flow rate (mL min^{-1}): 0.20<br>Focus flow rate (mL min^{-1}): 1.30<br>Cross flow rate (mL min^{-1}): 1.00<br>Injection time (min): 7.0<br>Transition time (min): 0.5	
**1st elution step**<br>Elution time (min): 35.0<br>Elution type: linear<br>Exponent: 1<br>Initial cross flow (mL min^{-1}): 1.00	**2nd elution step**<br>Elution time (min): 10.0<br>Elution type: constant<br>Exponent: 0<br>Initial cross flow (mL min^{-1}): 0.00

S.2. DLS study of different NPs sizes. The following tables show the hydrodynamic diameters of different AuNPs and AgNPs dispersions as function of dilution preparation time.

Table S3. Hydrodynamic diameters for AuNPs dispersions as a function of dilution preparation time.

NPs size (nm) / Time (h)	20	40	60	80
**0**	31.4	41.2	79.7	96.4
**24**	29.5	39.8	80.2	99.1
**48**	34.4	42.2	80.9	97.3
**72**	33.8	41.8	81.6	100.4

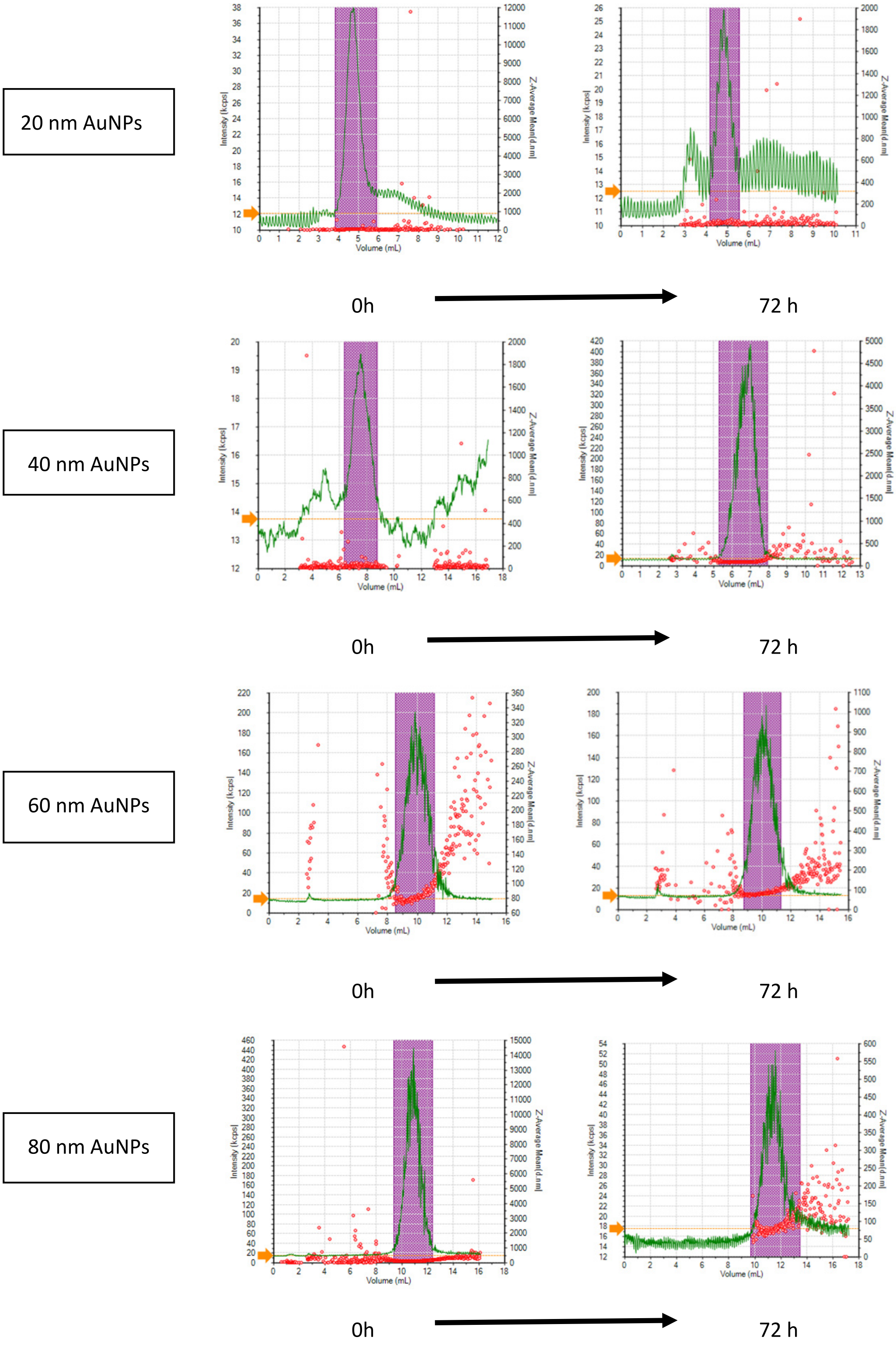

Figure S1. DLS spectra for AuNPs dispersions as a function of dilution preparation time.

Table S4. Hydrodynamic diameters for AgNPs dispersions as a function of dilution preparation time

NPs size (nm) / Time (h)	20	40	60
**0**	30.7	49.7	78.9
**3**	29.4	51.3	77.3
**24**	32.0	50.8	80.8
**72**	36.0	50.4	79.9

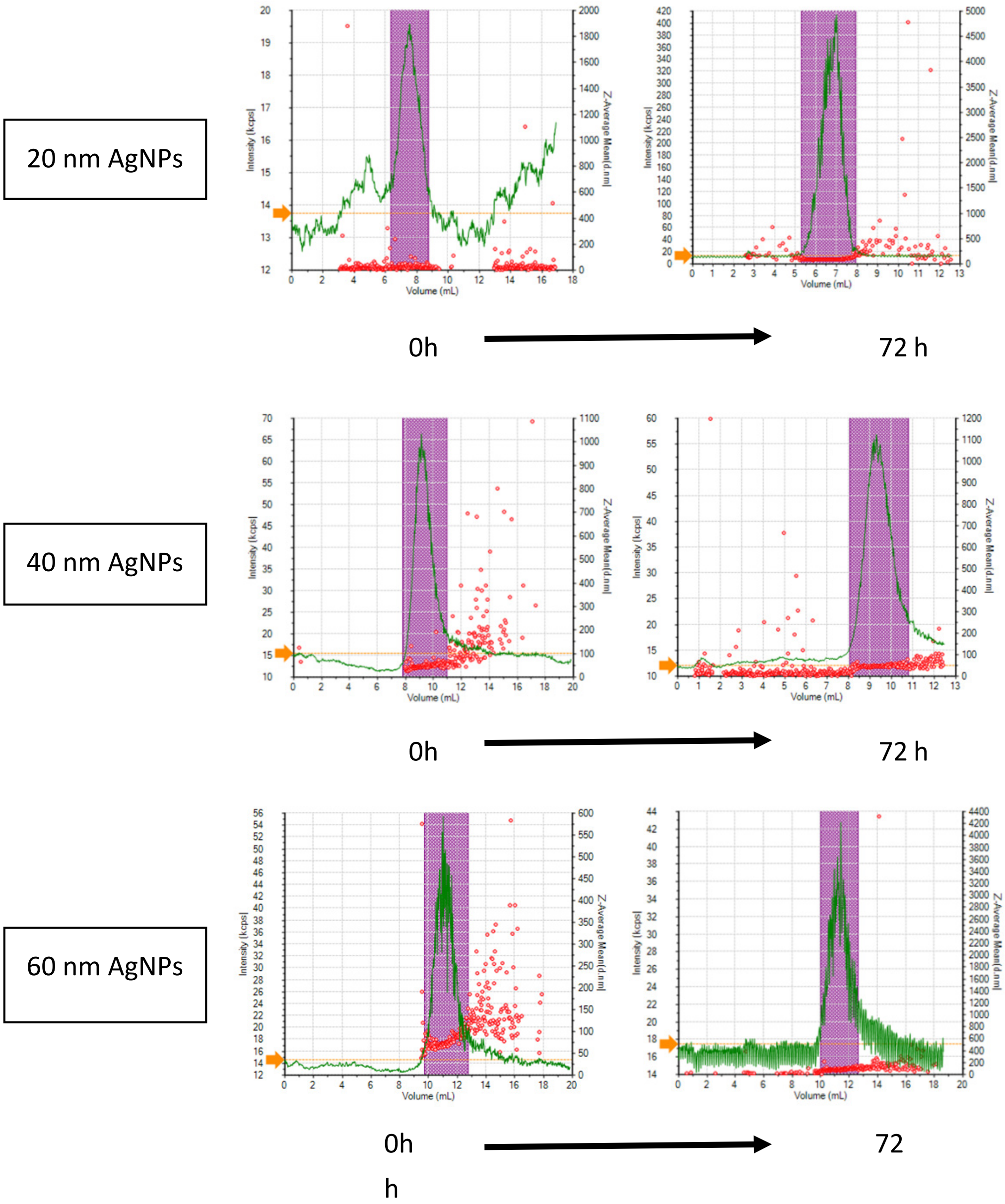

Figure S2. DLS spectra for AgNPs dispersions as a function of dilution preparation time.

S.3.

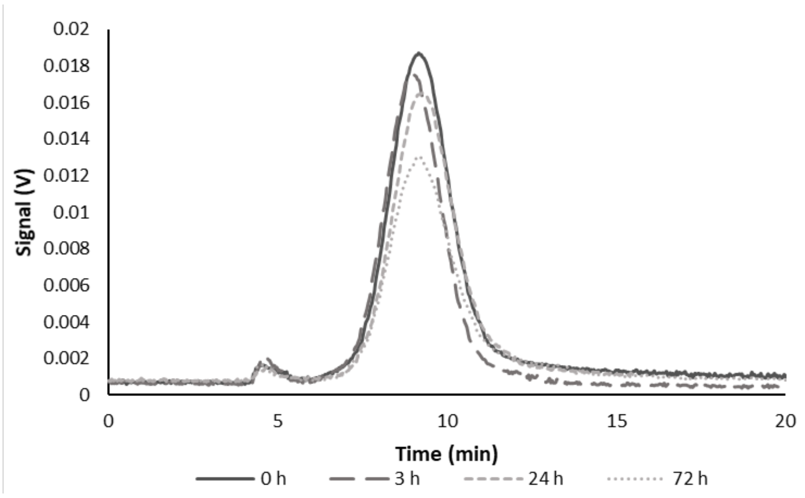

Figure S3. Study of AuNPs-PBS dispersions as function of dilution preparation time.

Funding: EU FEDER and the Gobierno de España MCIU-AEI (CTQ2017-90082-P), the Generalitat Valenciana (PROMETEO Program 2020/078) and EU-FEDER-Generalitat Valenciana (IDIFEDER/2018/049). L. Sanjuan-Navarro expresses his gratitude for the FPU-grant 17-01655 (MCIU-AEI).
